# Iron enhances reactive oxygen species generation and initiates neutrophil extracellular traps formation on the endothelium to exacerbate stroke

**DOI:** 10.1002/ccs3.70050

**Published:** 2025-10-03

**Authors:** Weijian Fan, Zebin Fang, Yuxiang Weng, Tianxiang Zhan, Kaiyuan Huang, Jianwei Pan, Renya Zhan

**Affiliations:** ^1^ Department of Neurosurgery The First Affiliated Hospital of Zhejiang University School of Medicine Hangzhou Zhejiang China

**Keywords:** ascorbic acid, endothelial cell, extracellular trap, Fe^2+^, MCAO, ROS

## Abstract

Endothelial‐derived reactive oxygen species (ROS), modulated by free iron levels, are key drivers of neutrophil extracellular traps (NETs) formation and contribute to cerebral ischemia/reperfusion (CI/R) injury. Targeting ROS and iron could possibly reduce NETs formation and mitigate stroke. NETs were predominantly initiated by hypoxia/reoxygenation (H/R) in endothelial cells (ECs), rather than in neutrophils. Silencing Fpn1 in ECs significantly reduced extracellular iron, suppressed ROS production, and inhibited NETs formation—effects that were reversed by supplementation with iron or hemin. Additionally, both vitamin C (Vc) and deferoxamine (DFOM) suppressed blood coagulation on Fpn1‐silenced ECs under H/R conditions. NETs formation on hypoxic ECs was further enhanced in the presence of co‐cultured red blood cells. In a mouse model of middle cerebral artery occlusion, combined treatment with Vc and DFOM synergistically reduced infarct size. Although Vc or DFOM alone reduced NETs formation, their combined use showed a weaker effect than DFOM alone, possibly because better blood flow increased neutrophil contact with the endothelium. In conclusion, endothelial ROS and iron play critical roles in regulating NETs formation during CI/R injury. Combined treatment with Vc and DFOM offers a promising therapeutic strategy to reduce stroke‐induced damage by modulating ROS levels and NETs formation.

## Graphical abstract

Under hypoxic conditions, iron from red blood cells and injured tissues is taken up by endothelial cells. During reoxygenation, this iron is exported via Fpn1, promoting NETs formation by enhancing ROS production through the Fenton reaction, ultimately exacerbating cerebral injury. Reducing ROS with vitamin C and chelating iron with deferoxamine effectively suppresses NETs formation and mitigates cerebral damage in stroke.

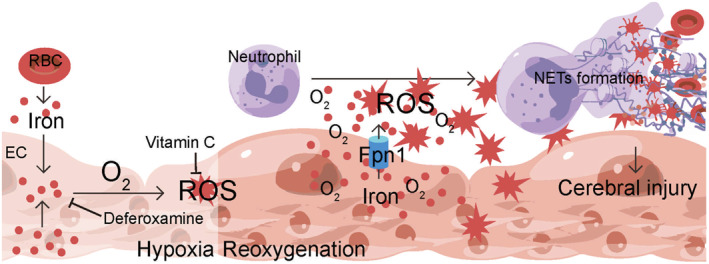

## 1 INTRODUCTION

Stroke, caused by a lack of blood supply or loss of vascular integrity in the brain, has become the second leading cause of death and the third leading cause of disability worldwide in recent years.^1^ The global burden of stroke has nearly doubled over the past 30 years, particularly in low‐ and middle‐income regions, and is expected to rise due to increasing populations and aging demographics in Southeast Asia, East Asia, and the Oceania super‐region.^2^ Ischemic stroke accounts for approximately 87% of all stroke cases, and nearly 90% of all strokes involve cerebral ischemia.^3^ Despite available treatments, the long‐term outcomes for stroke patients remain limited. Recombinant tissue plasminogen activator (rt‐PA), administered within 3 h of symptom onset, is considered the most effective treatment and is endorsed by the National Institute of Neurological Disorders and Stroke for its potential to improve functional outcomes. However, rt‐PA does not significantly improve survival compared to placebo.^4^ These limitations underscore the urgent need to develop new therapeutic strategies, particularly for the acute phase of stroke.

Neutrophil extracellular trap (NET) has emerged as a key contributor to neuronal injury following stroke. Neutrophils infiltrate the injured brain, where they become activated and release NETs, which subsequently damage the blood–brain barrier (BBB), inhibit neovascularization, induce neurological deficits, and exacerbate stroke prognosis.^5^ NETs are more abundant in cerebral thrombi than in coronary thrombi and increase with thrombus age, contributing to thrombolysis resistance.^6^ As a result, NETs presence has been positively correlated with disease severity and poor prognosis in ischemic stroke patients.^7^ Conversely, targeting neutrophils to reduce NETs formation has been shown to alleviate inflammation, improve neurological function, and enhance survival in stroke models.^8^ Although DNase I has been widely used in experimental settings to degrade NETs,^5^ there are currently no safe and effective NET‐targeting therapies available for clinical use.

Timely restoration of blood flow remains the primary therapeutic goal in stroke management.^9^ However, reperfusion following stroke often results in cerebral ischemia/reperfusion (CI/R) injury, which can exacerbate neuronal damage.^10^ One of the key mechanisms of CI/R injury is the generation of reactive oxygen species (ROS). Ischemia and hypoxia lead to energy failure in brain cells, resulting in disrupted glycolysis, ion imbalances, and mitochondrial damage.^11^ These events contribute to excessive ROS production during reperfusion,^12^ causing oxidative stress, ferroptosis, NETs formation, and vascular injury.^13^, ^14^ ROS scavenging has demonstrated neuroprotective effects and reduced CI/R injury in preclinical studies,^15^ suggesting that targeting ROS is a viable strategy to minimize neuronal damage after reperfusion.

The central nervous system primarily contains resident immune cells such as microglia in the brain parenchyma, and fewer CNS‐associated macrophages at brain‐vascular interfaces.^16^ Therefore, most NET‐forming neutrophils after stroke are derived from the bone marrow via circulation.^17^ Although some studies suggest that brain‐infiltrating neutrophils may originate from the brain borders,^18^ they are likely recruited through chemokine signaling involving CXCL2 positive cells.^19^ Regardless of their origin, the endothelium at the injury site serves as the first point of contact for circulating neutrophils, playing a critical role in their adhesion, localization, activation, and NETs formation. Peroxiredoxins (PRXs), particularly PRX4, are key antioxidant enzymes that eliminate up to 90% of intracellular H_2_O_2_.^20^ Endothelial‐specific deletion of PRX4 has been shown to worsen BBB disruption, increase microvascular inflammation, and exacerbate long‐term neurological deficits and brain atrophy in CI/R models.^21^ These findings highlight the importance of ROS scavenging in endothelial cells (ECs) and suggest its potential in preventing NETs formation.

ROS can be generated through multiple pathways, including NADPH oxidase (NOX), mitochondria, and the Fenton reaction. NOX has been identified as a major contributor to ROS production in ECs under hypoxia/reoxygenation (H/R) conditions.^22^ Although NETs can be induced by various stimuli, clinical strategies more commonly focus on ROS scavenging rather than direct NOX inhibition.^23^ Therefore, antioxidants and iron chelators are promising therapeutic candidates for targeting NETs formation during CI/R injury.

Based on these considerations, we hypothesized that endothelial ROS, regulated by iron availability, is a key driver of NETs formation during CI/R. To test this hypothesis, we investigated the role of iron and ROS with vitamin C (Vc) and deferoxamine mesylate (DFOM) intervention in NETs formation on ECs under H/R conditions, and evaluated the therapeutic effects of Vc and DFOM in a mouse model of middle cerebral artery occlusion and reperfusion (MCAO/R).

## 2 MATERIALS AND METHODS

### 2.1 Cell culture and transfection

Human umbilical vein ECs (HUVEC, FH1122, Fuheng Bio), human myeloid cell line (HL‐60, FH0101, Fuheng Bio), mouse brain angioendothelioma cells (bEnd.3, CL‐0598, Procell), and mouse aortic ECs (MAECs; FH0499, Fuheng Bio) were cultured in Dulbecco's Modified Eagle Medium (DMEM, GNM12800‐5, Genomcell) supplemented with 10% fetal bovine serum (FBS; 10100147, Gibco) and 1% penicillin‐streptomycin (GNM15140‐1, Genomcell). Cells that reached 90% confluence were passaged, or stored in frozen FBS. All cell were used in passages 6–10. For treatments, the seeded cells were incubated in 2% FBS when they reached about 80% confluence.

Fpn1 was silenced in mouse aortic endothelial cells (MAECs) using shRNA delivered via adeno‐associated virus (AAV) vectors (GenePharma). For transfection, 50 multiplicities of infection AAV were added to each well of a 6‐well plate in 8 μg/mL polybrene (SX50113, Shawnxin Bio) and incubated in serum‐free OPTI‐MEM medium (#31985070, Gibco) for 6 h. Cells were then cultured in complete medium for 48 h. Transfected cells were selected using 2 μg/mL puromycin (60209ES10, Yeason). Successful transfection was confirmed by assessing Fpn1 mRNA and protein levels. AAV‐packaged scrambled shRNA (sh‐NC) was used as a negative control.

### 2.2 Neutrophil isolation and NETs induction

Murine neutrophils were isolated from the femur and tibia bone marrow using the Neutrophil Isolation Kit (#130‐097‐658, Miltenyi Biotec) following the manufacturer's instructions. Isolated neutrophils were suspended in DMEM containing 2% FBS.

**FIGURE 1:**
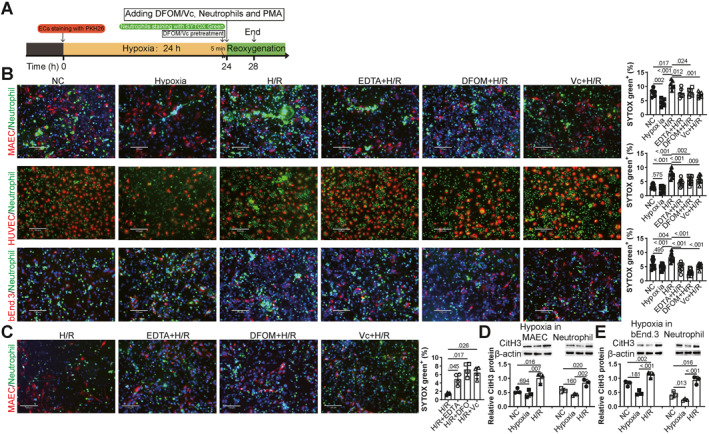
NETs are induced on ECs under H/R, dependent on ROS and iron. ECs were labeled with PKH26 (red) before reoxygenation, NETs were visualized using SYTOX Green, and SYTOX Green‐positive areas were analyzed using ImageJ. (A) The work flow of induction NETs and treatment in vitro. (B) MAECs (*N* = 6), HUVECs (*N* = 9), and mouse brain angioendothelioma (bEnd.3, *N* = 9) cells subjected to H/R were incubated with neutrophils to induce NETs. EDTA, DFOM, and Vc were co‐administered with neutrophils. (C) Mouse neutrophils subjected to H/R were incubated with normal MAECs to assess NETs formation. (D) NETs formation was confirmed by assessing CitH3 protein expression in both MAECs and neutrophils after H/R, *N* = 3. (E) NETs formation was confirmed in bEnd.3 cells and neutrophils after H/R, *N* = 3. *p* < 0.05 was considered statistically significant, as determined by one‐way ANOVA followed by Tukey's multiple comparisons test. DFOM, deferoxamine mesylate; ECs, endothelial cells; EDTA, ethylenediaminetetraacetic acid; H/R, hypoxia/reoxygenation; HUVECs, human umbilical vein ECs; MAECs, mouse aortic endothelial cells; NETs, neutrophil extracellular traps; ROS, reactive oxygen species; Vc, vitamin C.

The workflow for induction of NETs were illustrated in Figure 1A. To visualize NETs formation on ECs, MAECs were seeded in 24‐well glass‐bottom plates to reach about 80% confluence. Then the culture medium was changed to 2% FBS, and the ECs were stained with PKH26 Red Fluorescent Cell Linker (5 μM, HY‐D1451, MCE). Hypoxia was induced by covering the plates with PARAFILM® M membrane and reoxygenation was achieved by directly removing this membrane.^24^ Successful induction of hypoxia was confirmed by increased HIF‐1α mRNA. To validate the reliance of hypoxia induction, bEnd.3 cells were maintained in a 1% O_2_, 5% CO_2_, and 94% N_2_ chamber (MIC‐101, Glord). NETs were induced on ECs that underwent hypoxia for 24 h and upon reoxygenation, using neutrophils (6 × 10^5^ cells/mL) labeled with SYTOX Green (5 μM, S7020, Invitrogen).^25^ NETs were also induced using 100 nM phorbol 12‐myristate 13‐acetate (PMA; S1819, Beyotime) for 4 h, with or without co‐treatment of the following agents in DMEM: 2 mM ethylenediaminetetraacetic acid (EDTA, 0.5 M stock in DMSO, E885215, Macklin), 50 μM DFOM (50 μM in DMSO, D302525, Aladdin), 3 mM Vc (3 mM in DMEM, A103539, Aladdin), Fresh red blood cells (RBCs, 10^7^), 50 μM FeSO_4_ (50 mM in DMEM, F710256, Aladdin), and 50 μM hemin (50 mM in DMSO, HY‐19424, MCE). SYTOX Green and DAPI (#D1306, Invitrogen) were added 5 min before imaging under a Nikon Ni‐U fluorescence microscope.

### 2.3 Biochemical assays

Cellular ROS levels were assessed using DCFH‐DA (10 μM; S0033S, Beyotime) according to the manufacturer's instructions. Cells were incubated for 40 min before observation under a fluorescence microscope.

Total iron (Fe^2+^ + Fe^3+^) content was measured using an iron assay kit (ab83366, Abcam). Briefly, 5 μL of iron reducer and 5 μL of assay buffer were added to each well of a 96‐well plate containing MAECs.

Clotting time was measured by adding fresh whole blood (200 μL) to a 96‐well plate pre‐cultured with MAECs or their homogenates. Clotting was monitored every 60 s by gently tilting the plate.

### 2.4 Real‐time quantitative polymerase chain reaction (qPCR)

Total RNA was extracted from treated MAECs using the RNeasy Kit (DP419, Tiangen Biotech) and reverse‐transcribed with oligo‐dT primers and PrimeScript II RTase (#RR055A, Takara). Quantitative PCR (qPCR) was conducted using SYBR Green Super Mix (#1725121, Bio‐Rad). The following primer pairs were used: Fpn1 (forward, 5′‐CTCCA ACCCG CTCCC ATAAG‐3′; reverse, 5′‐TCATG ACACC AGGCG TTCTC‐3′) GAPDH (forward, 5′‐TGGTG AAGCA GGCAT CTGAG‐3′; reverse, 5′‐TGAAG TCGCA GGAGA CAACC‐3′). Relative expression levels were calculated using the 2^−ΔΔCt^ method.

### 2.5 Western blotting

Proteins were extracted from MAECs using radio immunoprecipitation lysis buffer (P0013B; Beyotime) on ice for 30 min. Lysates were centrifuged at 4°C for 15 min, and protein concentration was determined via bicinchoninic acid assay. Equal amounts (20 μg) of protein were separated by 10% sodium dodecyl sulfate‐polyacrylamide gel electrophoresis and transferred onto polyvinylidene fluoride membranes (Millipore). Membranes were blocked with 5% skim milk in tris buffered saline with tween 20 for 30 min and incubated overnight at 4°C with primary antibodies: Anti‐VE Cadherin antibody (1:1000, ab282277, Abcam) and β‐actin Ab (1:5000, AB0011, Abways). After washing, membranes were incubated with secondary antibodies goat anti‐rat IgG (1:5000, ab205720, Abcam) or goat anti‐mouse IgG (1:10,000, AB0102, Abways). Signals were detected using enhanced chemiluminescence reagents (MA0186, MeilunBio).

### 2.6 Animal experiments

All animal experiments complied with GB/T 35823‐2018 and were approved by the Ethics Committee of the First Affiliated Hospital, College of Medicine, Zhejiang University (approval number: 2017SDKS092, approval date: February 15, 2017). Male C57BL/6J mice (6–8 weeks old; SIPPR‐BK) were challenged with CI/R, and randomly assigned to four groups: CI/R, Vc, DFOM, and Vc + DFOM (*n* = 9 per group).

CI/R was induced by MCAO/R after 12 h fasting. Cerebral blood flow was monitored to confirm both occlusion and reperfusion with a laser Doppler probe attached above the skull. Mice were anesthetized with isoflurane, and the right common carotid artery (CCA), internal carotid artery (ICA), and external carotid artery (ECA) were exposed. The ECA and CCA were ligated, and the ICA was clamped. A “V”‐shaped incision was made in the CCA, and a suture plug was inserted ∼18–20 mm to occlude the middle cerebral artery.

After 2 h of ischemia, mice received intraperitoneal injections of Ascorbic acid (200 mg/kg) or DFOM mesylate (100 mg/kg). Both drugs were administered in 200 μL volume in saline, 10 min before reoxygenation. The CI/R model group were given 200 μL saline. Reperfusion was initiated by removing the suture, by which chronic hypoperfusion rather than full reperfusion was achieved. After 24 h, mice were euthanized using CO_2_ followed by cervical dislocation. Whole brains were collected. Three brains per group were used for triphenyltetrazolium chloride (TTC) staining; the remainder were halved, one half was snap‐frozen in liquid nitrogen and stored at −80°C, the other was fixed in 4% paraformaldehyde (PFA).

### 2.7 Tissue staining

For TTC staining, the mice brains were rinsed in cold phosphate buffered saline and frozen at −20°C for 10 min. Coronal sections (2 mm thick, five slices per brain) were prepared and incubated with 2% TTC Staining Solution (C0652, Beyotime) for 10 min, and fixed with 4% PFA for 10 min after washed out thrice. The sections were photographed with a camera, and stored at −80°C. The infarct volume was analyzed with ImageJ and calculated by the sum of the infarct area multiplied by 2 mm of each slice.

For immunofluorescence staining, the PFA‐fixed brain tissues were paraffin‐embedded and sectioned at 16 μm. After rehydration, sections were blocked in 1% BSA/5% goat serum, followed by overnight incubation at 4°C with primary antibodies against CitH3 and CD31 (antibody details not provided). Secondary antibodies included Alexa Fluor 488‐conjugated goat anti‐mouse IgG (#A28175, Invitrogen). Nuclei were counterstained with DAPI (#C1002, Beyotime). Stained sections were imaged using a Nikon Ni‐U microscope.

### 2.8 Transcriptome analysis

Transcriptome sequencing was performed by Novogene Co. Ltd. on brain tissues from three mice per group, following their standard protocols. For bioinformatics analysis, reads containing adapters, poly‐N sequences, and low‐quality reads were removed from the raw data. All downstream analyses were conducted using the resulting clean data.

As gene expression profiles were relatively similar across groups, all differentially expressed genes (DEGs, *p* ≤ 0.05, as listed in Table S1, part 1) were included in the Gene Set Enrichment Analysis (GSEA). GSEA was performed based on both Gene Ontology (GO) terms (Table S1, parts 2–4) and Kyoto Encyclopedia of Genes and Genomes (KEGG) pathways (Table S1, parts 5–7). The top 30 significantly enriched pathways from each comparison—Vc versus CI/R, DFOM versus CI/R, and Vc + DFOM versus CI/R—were combined, and the enrichment of these pathways across all three comparisons was visualized in a dot plot.

### 2.9 Statistical analysis

Data are presented as mean ± standard deviation. One‐way ANOVA followed by Tukey's multiple comparisons test or unpaired *t*‐tests were performed using GraphPad Prism 9.5 (GraphPad Software). A *p*‐value <0.05 was considered statistically significant.

## 3 RESULTS

### 3.1 NETs formation during reoxygenation is dependent on iron and ROS

NETs formation was initially observed on hypoxic ECs, including MAECs, HUVECs and bEnd.3 cells. Upon PMA stimulation for 4 h, NETs levels were reduced under hypoxia compared to the normoxic control but were significantly increased after reoxygenation. This reoxygenation‐induced NETs formation was attenuated by EDTA, DFOM, and Vc treatment (Figure 1B). Similarly, hypoxia was induced in neutrophils (Figure 1C). In contrast to ECs, few NETs were observed during reoxygenation in neutrophils, and NETs formation was increased by EDTA, DFOM, and Vc. Since CitH3 immunofluorescence staining was not stably detectable with us, NETs were primarily assessed using SYTOX Green. Comparable trends observed in CitH3 protein levels confirmed the reliability of SYTOX Green as a substitute (Figure 1D,E). These results indicate that NETs are initially formed on hypoxic ECs during reoxygenation, and this process is dependent on iron and ROS.

### 3.2 NETs formation is regulated by extracellular iron and ROS

**FIGURE 2:**
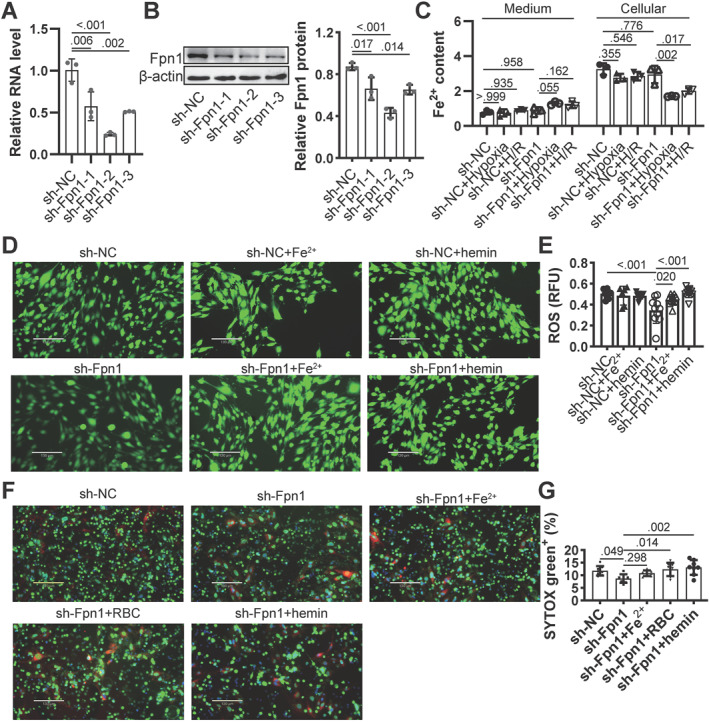
Impact of iron transport in NETs formation on MAECs under H/R conditions. (A, B) Fpn1 was silenced in MAECs to inhibit iron export, confirmed by qPCR and western blotting, *N* = 3. (C) Iron concentrations were measured in the culture medium and cell lysates of Fpn1‐silenced MAECs under H/R, *N* = 3. (D) ROS levels were assessed in transfected MAECs treated with iron or hemin. (E) ROS levels were quantified using ImageJ, *N* = 10. (F) NETs formation on MAECs under H/R was evaluated, with or without treatment with iron, RBCs, or hemin. (G) NETs levels were quantified using ImageJ, *N* = 8. *p* < 0.05 was considered statistically significant by one‐way ANOVA with Tukey's multiple comparisons test. H/R, hypoxia/reoxygenation; MAECs, mouse aortic endothelial cells; NETs, neutrophil extracellular traps; qPCR, quantitative PCR; RBCs, red blood cells; ROS, reactive oxygen species.

To determine the contribution of intracellular iron to NETs formation, Fpn1 was silenced to inhibit iron efflux. Silencing efficiency was confirmed by qPCR and western blot (Figure 2A,B). Fpn1 silencing did not significantly alter intracellular iron levels under either hypoxia or post‐reoxygenation conditions. However, extracellular iron levels were notably reduced in Fpn1‐silenced cells under both conditions (Figure 2C). RBCs and hemin were introduced as they can act as a possible source of iron under hypoxia. In sh‐NC cells, the addition of iron or hemin did not increase ROS levels, whereas in sh‐Fpn1 cells, ROS was reduced and subsequently restored by iron or hemin supplementation (Figure 2D,E). NETs formation was slightly reduced on Fpn1‐silenced ECs, but this effect was reversed by different form of extracellular iron, including co‐culture with FeSO_4_, RBCs or hemin (Figure 2F,G). These findings suggest that intracellular iron from ECs enhances ROS generation and NETs formation.

**FIGURE 3:**
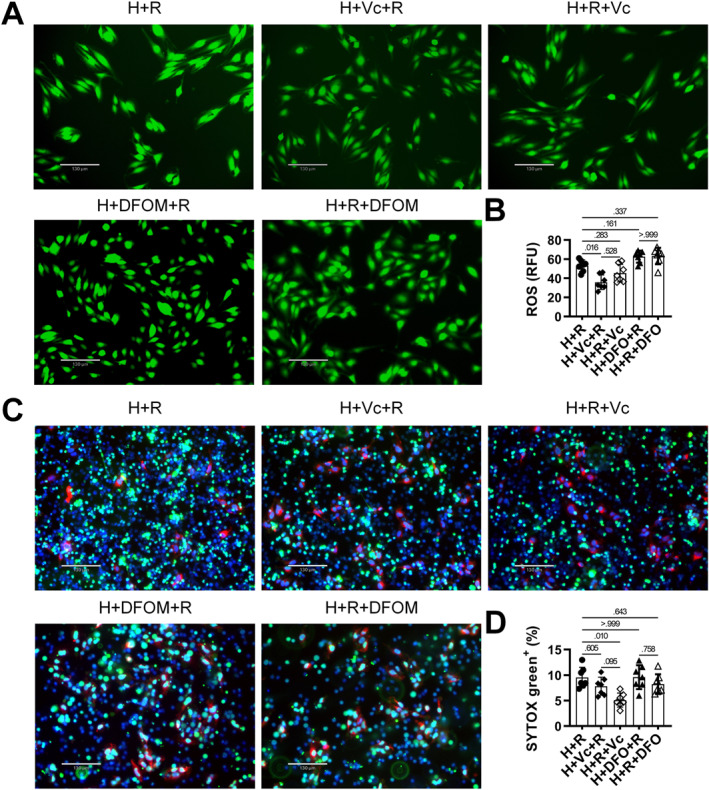
Effects of pretreated and post‐added Vc on ROS and NETs formation under H/R. Vc was either added 5 min prior to reoxygenation (pretreated) or immediately at reoxygenation (post‐added). (A) ROS content in MAECs. (B) ROS intensity was quantified using ImageJ, *N* = 7. (C) NETs formation following different Vc or DFOM treatment timings. (D) NETs formation was quantified using ImageJ, *N* = 7. *p* < 0.05 was considered statistically significant by one‐way ANOVA with Tukey's multiple comparisons test. DFOM, deferoxamine mesylate; H/R, hypoxia/reoxygenation; MAECs, mouse aortic endothelial cells; NETs, neutrophil extracellular traps; ROS, reactive oxygen species; Vc, vitamin C.

To evaluate optimal timing for antioxidant and iron chelator administration in reducing NETs and mitigating CI/R, Vc or DFOM was added either 5 min before reoxygenation,^26^ or together with neutrophils immediately afterward. Pretreatment with Vc significantly reduced ROS levels, whereas Vc added post‐reoxygenation and DFOM showed negligible effects (Figure 3A,B). Interestingly, Vc pretreatment was less effective in reducing NETs compared to post‐reoxygenation Vc addition (Figure 3C,D), and DFOM did not significantly affect NETs formation. These results suggest that extracellular iron and baseline ROS synergistically enhance ROS levels, thereby promoting NETs formation during reoxygenation.

### 3.3 Hypoxic RBCs enhance ROS generation and promote NETs formation during reoxygenation

**FIGURE 4:**
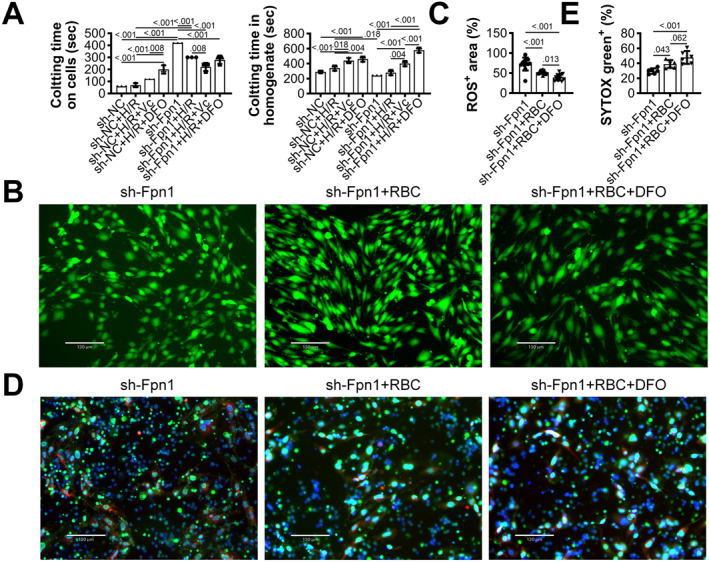
RBCs enhance ROS generation and NETs formation under H/R. (A) Coagulation of whole blood on the surface of intact or homogenized MAECs after 24 h hypoxia and 4 h reoxygenation, *N* = 3. (B) ROS levels in sh‐Fpn1‐transfected MAECs treated with RBCs prior to hypoxia and with DFOM during reoxygenation, *N* = 12. (C) ROS intensity quantified by ImageJ. (D) NETs formation on sh‐Fpn1‐transfected MAECs under the same treatment conditions. (E) NETs were quantified using ImageJ, *N* = 6. *p* < 0.05 was considered statistically significant by one‐way ANOVA with Tukey's multiple comparisons test. DFOM, deferoxamine mesylate; H/R, hypoxia/reoxygenation; MAECs, mouse aortic endothelial cells; NETs, neutrophil extracellular traps; RBCs, red blood cells; ROS, reactive oxygen species.

Considering the presence of residual blood in hypoxic regions, we explored the interaction between blood and ECs under hypoxia and reoxygenation. In hypoxic sh‐NC‐transfected MAECs, reoxygenation in the presence of whole blood led to rapid coagulation within approximately 1 min, which was delayed by Vc or DFOM treatment (Figure 4A). Coagulation was significantly prolonged in Fpn1‐silenced cells but was reduced upon treatment with Vc or DFOM. In cell homogenate experiments, coagulation was inhibited in both sh‐NC and sh‐Fpn1 groups. In sh‐Fpn1 MAECs co‐cultured with RBCs prior to hypoxia, ROS levels were slightly reduced, with no statistical significance, but significantly decreased when DFOM was added during reoxygenation (Figure 4B,C). Interestingly, NETs formation showed an opposite trend: co‐cultured RBCs during hypoxia significantly increased NETs formation, which was further enhanced by DFOM (Figure 4D,E). These results highlight the promotive effect of RBCs on NETs formation under H/R conditions.

### 3.4 Vc and DFOM reduce NETs to alleviate CI/R injury in mice

**FIGURE 5:**
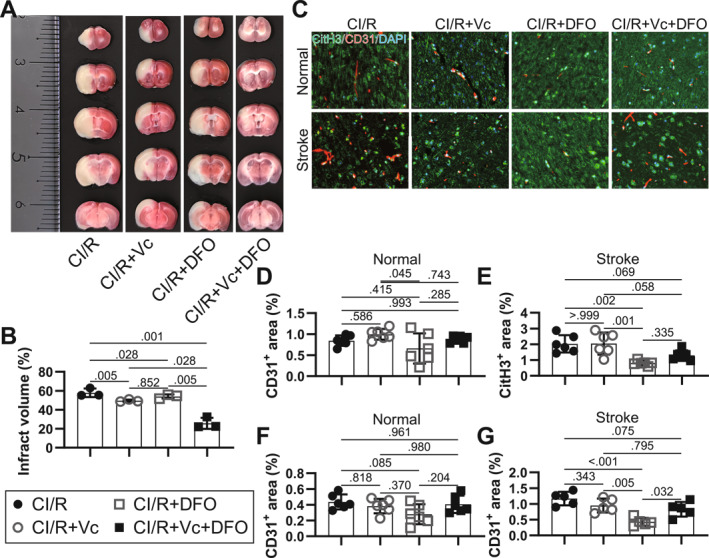
Protective effects of DFOM and Vc in MCAO/R mice via inhibition of NETs formation. MCAO/R was induced in C57BL/6c mice to model CI/R with 2 h ischemia, with DFOM and Vc administered intraperitoneally 10 min before reoxygenation. Brain tissue was collected 24 h after reoxygenation. (A) Infarct areas were visualized by TTC staining. (B) Infarct volume was calculated from TTC‐negative areas using ImageJ, *N* = 3. (C) Immunofluorescence staining for CD31 (capillaries) and CitH3 (NETs) in mouse brain tissue. (D, E) NETs formation in the normal and injured brain regions, respectively, was quantified with ImageJ, *N* = 6. (F, G) Capillary density in the normal and injured brain regions, respectively, was analyzed using ImageJ, *N* = 6. *p* < 0.05 was considered statistically significant by one‐way ANOVA with Tukey's multiple comparisons test. CI/R, cerebral ischemia/reperfusion; DFOM, deferoxamine mesylate; MCAO/R, middle cerebral artery occlusion and reperfusion; NETs, neutrophil extracellular traps; TTC, triphenyltetrazolium chloride; Vc, vitamin C.

The in vivo effects of Vc and DFOM were evaluated in a mouse model of CI/R. Three mice died of CI/R modeling, and 6 died of the failed attempts to achieve intra‐arterial drug delivery. TTC staining showed that the average infarct volume of the CI/R group was 57.87%. Treatment with either Vc or DFOM alone had limited impact on reducing infarct volume (49.54% and 54.01%, respectively), whereas their combined use led to a significant reduction of 25.70% (Figure 5A,B), which is a typical synergic effect. NETs and small vessel presence were visualized via CitH3 and CD31 immunofluorescence. Co‐localization of CitH3 and CD31 was primarily observed in the unaffected hemisphere (Figure 5C). The intensity of CitH3 remained relatively stable across groups in this region, whereas CD31 expression was reduced in the DFOM group (Figure 5D,E), with this effect partially reversed in the co‐treatment group. In the injured hemisphere, both CitH3 and CD31 signals were significantly altered in response to DFOM but not Vc treatment (Figure 5F,G). The trends of CitH3 and CD31 were largely consistent across tissues, suggesting that NETs formation is closely linked to endothelial status and may account for the reduced NETs inhibition observed in the cotreatment group. The results support that the antioxidants Vc with iron chelator DFOM can reduce NETs and synergize to alleviate CI/R injury in mice.

**FIGURE 6:**
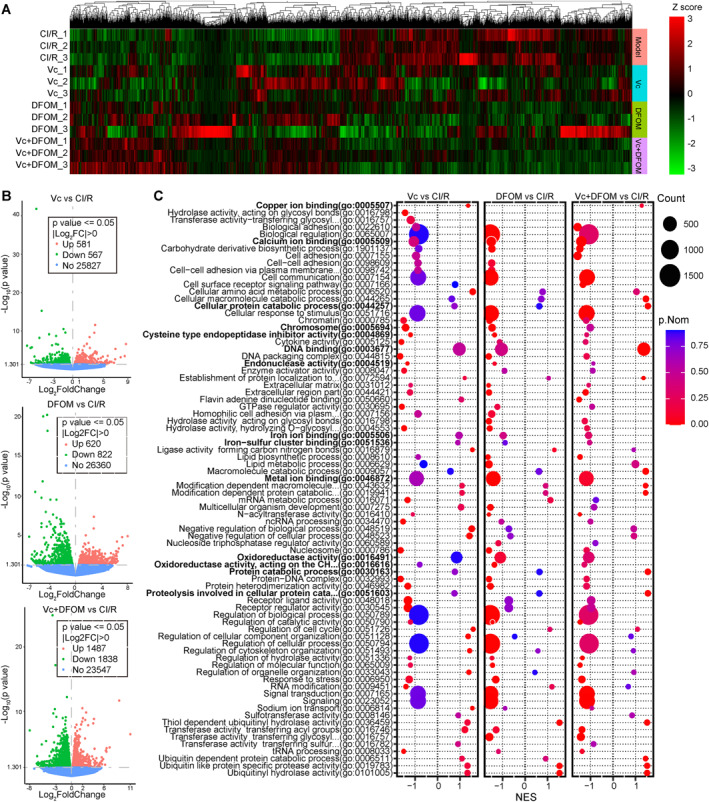
Bioinformatics analysis of mouse brain tissues. (A) Heat map of the DEGs. (B) Volcano plots showing differential gene expression for Vc versus CI/R, DFOM versus CI/R, and Vc + DFOM versus CI/R. (C) GSEA was conducted using GO terms. The top 50 most significantly enriched terms and several oxidative stress‐ and iron metabolism‐associated GO terms for each comparison were identified, and their enrichment across comparisons is displayed. CI/R, cerebral ischemia/reperfusion; DEGs, differentially expressed genes; DFOM, deferoxamine mesylate; GO, gene ontology; GSEA, gene set enrichment analysis; Vc, vitamin C.

Transcriptome analysis was performed on mouse brain tissues, and heatmap of the DEGs was shown in Figure 6A. Overall, the effects of MCAO/R were reversed by treatment. Genes that were highly expressed under MCAO/R but downregulated following treatment were NETs formation associated, and can be enriched in pathways such as pathways in cancer, cytokine–cytokine receptor interaction, complement and coagulation cascade, leukocyte transendothelial migration, and platelet activation. In contrast, the treatment upregulated DEGs were primarily associated with circadian rhythm, mTOR signaling, and autophagy. A total of 581 upregulated and 567 downregulated genes were identified in the Vc versus CI/R comparison; 620 upregulated and 822 downregulated genes in DFOM versus CI/R; and 1487 upregulated and 1838 downregulated genes in Vc + DFOM versus CI/R (Figure 6B). GSEA revealed that most enriched GO terms were downregulated (Figure 6C). Specifically, cellular macromolecule catabolic process, DNA binding, and protein catabolic process were insignificant with Vc or DFOM treatment group, whereas significantly activated in Vc + DFOM group. On the other hand, chromosome, cysteine‐type endopeptidase inhibitor activity, and endonuclease activity were significant in Vc or DFOM treatment group while turned insignificant in Vc + DFOM group. Iron–sulfur cluster binding and iron ion binding were not significantly enriched in Vc or DFOM treatment group, and their significance lowered in Vc + DFOM group. Oxidoreductase activity was significantly inhibited in DFOM group while turned insignificant in Vc + DFOM group.

**FIGURE 7:**
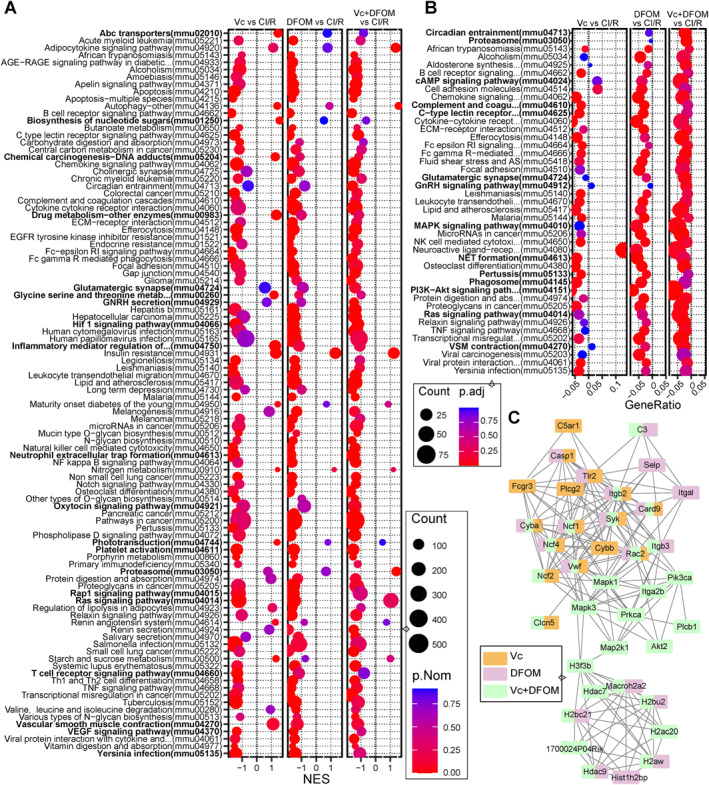
KEGG and PPI analysis of the DEGs. (A) GSEA was conducted with all (*p* < 0.05) using the KEGG datasets. The top 50 significantly enriched pathways, and their enrichment across comparisons is displayed. (B) KEGG enrichment was applied with the most significantly altered genes, the top 20 significantly enriched pathways, and their enrichment across comparisons. (C) PPI for genes enriched in the NET formation pathway. DEGs, differentially expressed genes; GSEA, gene set enrichment analysis; KEGG, Kyoto Encyclopedia of Genes and Genomes; NET, neutrophil extracellular trap; PPI, protein–protein interaction.

GSEA for KEGG was also applied (Figure 7A). The result suggested that circadian entrainment, glutamatergic synapse, GnRH secretion, oxytocin signaling pathway, and proteasome were not enriched in Vc or DFOM group while significantly enriched in Vc + DFOM group. Glycine serine and threonine metabolism, Ras signaling pathway, Rap1 signaling pathway, T cell receptor signaling pathway, *Yersinia* infection, and vascular endothelial growth factor signaling pathway were significantly enriched in Vc or DFOM group while turn insignificant in Vc + DFOM group. Specifically, Hif‐1 signaling pathway was enriched across these treatments, supporting the presence of reduced oxidative stress. NET formation was also significantly inhibited across treatments, as well as its associated pathways such as Fc gamma R‐mediated phagocytosis, platelet activation, and complement and coagulation cascades. Consistent with the tissue staining results, NETs‐associated pathways were more significantly enriched in the DFOM group than in the Vc + DFOM group. Similar outcomes were observed with simple KEGG enrichment (Figure 7B). The protein–protein interaction for DEGs enriched in the NET formation pathway were analyzed (Figure 7C), and key NETs‐related genes such as Cyba, Ncf1, Ncf4, Vwf, Syk, Itgb2, and Card9 were present in all these comparisons. Additionally, C5ar1, Fcgr3, Plcg2 and Cybb was only downregulated in Vc group; Itgal, Hist1h2bp, and Selp were only reduced in DFOM group; several other genes were only lowered in Vc + DFOM group, including Mapk1, Mapk3, Itga2b, Prkca, Hdac7, and Akt2. These findings suggest that both Vc and DFOM suppress NETs formation following stroke; however, this effect appears to be partially diminished when the two treatments are combined, even though Vc and DFOM clearly act synergistically to alleviate stroke.

## 4 DISCUSSION

NETs have emerged as key contributors to stroke pathogenesis in recent years. Among immune cells, neutrophils are the first to be recruited to the site of ischemic injury. These infiltrating cells aggravate brain damage by producing ROS, proteases, and lipocalin‐2, as well as by forming NETs.^27^ After stroke, the injured brain releases large amounts of HMGB1, which activates the RAGE and TLR4 pathways, transmitting danger signals to surrounding tissues. Additionally, the JAK‐STAT signaling pathway is markedly upregulated, promoting inflammation and edema. Both HMGB1 and JAK/STAT signaling are potent inducers of NETs.^28^ Other dysregulated factors in stroke, such as neuronal cold‐inducible RNA‐binding protein, have also been shown to induce NETs formation.^27^ Generally, NETs formation is considered to be dependent on neutrophil‐derived ROS.^29^ However, ROS can also be robustly produced by ischemic brain tissue, particularly through phospholipase A2 activation and lipid peroxidation.^30^ The influence of tissue‐derived ROS on NETs formation has remained unclear. In this study, we confirmed the pro‐NETs effect of neuronal ROS by observing NETs on the surface of hypoxic ECs. We further demonstrated that iron enhances ROS production, and that targeting iron and ROS with DFOM and Vc effectively reduced NETs formation and alleviated CI/R injury in a mouse MCAO model.

Neutrophils become activated in response to damage‐associated molecular patterns and are recruited to inflamed tissues by rolling and adhering to the endothelium, followed by transmigration.^31^ Endothelial dysfunction is common across stroke subtypes and significantly worsens clinical outcomes.^32^ Damaged ECs are sufficient to induce NETs formation,^25^, ^33^ and are also highly vulnerable to NETs, which can disrupt endothelial barrier integrity and induce cell death.^27^, ^34^, ^35^ A vicious cycle may result, wherein NETs and EC activation amplify each other, contributing to thrombosis and secondary cerebral injury.^31^, ^34^ Our findings support that the endothelium as the primary site for NETs formation following stroke reperfusion can serve as a valuable therapeutic target. We observed that NETs were more prominently induced by H/R‐treated ECs compared to neutrophils, highlighting the critical role of ECs in driving NETs formation during reperfusion. Stroke‐associated ferroptosis, characterized by lipid peroxidation and iron accumulation, facilitates the export of iron and extracellular ROS production.^13^ In the iron‐rich post‐stroke microenvironment, ROS can be readily generated via Fenton reactions, promoting ferroptosis and necroptosis in neuronal cells.^36^ Fpn1 is the only known cellular iron exporter. Endothelial‐specific Fpn1 deletion in stroke models has been shown to reduce cerebral iron and ROS levels, suppressing ferroptosis in the acute phase. However, long‐term Fpn1 deletion impaired recovery by promoting glial proliferation and hindering repair.^37^ These findings suggest that directly targeting extracellular iron during the acute phase, rather than targeting Fpn1 itself, may offer therapeutic benefits. In our study, Fpn1 knockdown reduced ROS and NETs formation, which could be restored by iron or hemin supplementation. Both DFOM and Vc effectively reduced ROS and inhibited NETs formation in vitro, especially when administered prior to reoxygenation. These results support the concept that extracellular iron and baseline ROS synergistically amplify oxidative stress and promote NETs formation during reoxygenation.

Ischemic stroke is driven by intravascular coagulation within cerebral vessels. NETs can interactively promote platelet activation and coagulation, thereby exacerbating stroke, enhancing atherosclerosis and inflammation, and contributing to resistance against rt‐PA thrombolysis.^28^ During H/R injury, ROS production and NETs formation are also influenced by residual blood components. Erythrocyte lysis begins within minutes after stroke onset and persists for days, releasing hemoglobin, oxidized hemoglobin, free heme, and iron. These iron forms can penetrate brain tissue, promoting toxicity, oxidative stress, inflammation, and glutamate‐mediated excitotoxicity.^38^ Our results further support the role of erythrocytes in NETs formation during CI/R, particularly in contused regions. In vitro, ECs co‐cultured with RBCs exhibited partial resistance to hypoxia, and ROS generation was significantly reduced with DFOM treatment. However, NETs formation was enhanced in these co‐cultures, and further increased with DFOM. This paradoxical effect may be due to oxygen released from RBCs or the retained catalytic activity of iron‐DFOM complexes. Even below normal blood glucose level to reach 0.1 mM, ECs can stay in a low oxygen consumption rate, which is necessary for oxygen transport.^39^ The RBCs in our treatments can thus act as an oxygen pool for ECs under hypoxia. On the other hand, DFOM can reduce peroxidation inhibited ROS generation upon any concentration.^40^ Treatment of 5 mM DFOM, a much higher level than that in this study, totally abolished the oxidative damage induced by oxygen glucose deprivation followed by reoxygenation (OGD/R) in bEnd.3 cells.^41^ These can explain the enhanced inhibition of ROS with incubation of RBCs. Iron chelation by DFOM was also reported to promote NETs formation in human blood‐derived neutrophils, which is dependent on NADPH and peptidyl‐arginine‐deiminase 4 (PAD4).^42^ The enhancement of NETs formation with DFOM and RBCs treatment can thus be induced by the chelation of iron from the RBCs, which is a rich pool of iron. Nevertheless, the NETs induction ability of DFOM and RBCs in vitro was moderate, and the obvious inhibition of NETs by DFOM was further confirmed in CI/R mice.

Both Vc and DFOM have been previously reported to mitigate CI/R injury and reduce NETs formation. For instance, systemic administration of Vc improved infarct volume, BBB integrity, and neuronal survival in MCAO rats, correlating with reduced ROS/reactive nitrogen species and inflammation.^43^ Vc alone or in combination with other antioxidants has been shown to suppress LPS‐induced NETs formation in vitro in a dose‐dependent manner.^44^ Iron overload drives both ferroptosis and necroptosis, and DFOM has demonstrated protective effects against these processes in H/R models.^36^ Elevated serum iron and heme in patients with sickle cell disease also promote NETs formation and inflammation, which can be reduced by DFOM treatment.^45^ DFOM acts as a siderophore, scavenging iron and preventing ROS generation in neutrophils.^46^ However, DFOM has also been reported to promote NETs formation under certain conditions, potentially due to residual ROS‐generating capacity of chelated iron complexes.^47^ The effects of Vc and DFOM were further validated in vivo through histological staining and bioinformatics analysis. Strikingly, Vc and DFOM only modestly reduced infarct volume, and the infarct volume was markedly reduced by co‐administration, exhibiting as a “synergistic effect.” DFOM significantly inhibited NETs formation in MCAO mice through histological staining. However, NETs inhibition was less pronounced in Vc + DFOM group compared to that in DFOM group, though NETs formation was significantly inhibited with either Vc or DFOM alone as indicated with bioinformatics. The result suggests that Vc and DFOM may have other interactive effects in improving CI/R, such as the pathways that enhanced or abolished in the cotreatment group. The effects of Vc and DFOM can be complementary despite their inhibition on NETs formation did not consistently demonstrate stronger inhibition. For example, vascular smooth muscle (VSM) contraction was not significantly enriched in either the Vc or DFOM groups, yet it was significantly inhibited in the cotreatment group according to KEGG enrichments. This may explain some part of the reduced inhibition of NETs formation in the cotreatment group, as reduced VSM contraction may enhance the recanalization after ischemia as supported by the markedly reduced infarct volume, which exposes more injury ECs to provide a larger interface for NETs forming. Importantly, the trends of NETs formation indicated by CitH3 were highly consistent with the endothelial marker CD31, supporting a close link between NETs and endothelial status. CD31 is known to decrease under hypoxia;^48^ in our study, it was unaffected by Vc but reduced by DFOM. In the cotreatment group, CD31 levels were comparable to those in the Vc group, likely reflecting reduced hypoxia. Since CD31 promotes neutrophil migration to sites of inflammation,^49^ it may also contribute to NETs formation through enhanced interactions between neutrophils and injured neurons.

This study confirms the therapeutic potential of targeting ROS to suppress NETs formation and mitigate CI/R injury, both through direct ROS scavenging by antioxidants (Vc) and by preventing ROS generation via the Fenton reaction using iron chelators (DFOM). However, certain limitations should be acknowledged. The H/R model used in cultured ECs may not fully replicate the complex in vivo interactions among ECs, iron, ROS, and neutrophils during stroke. For MCAO model, chronic hypoperfusion rather than full reperfusion was achieved with filament withdrawal. This may well reflect the stroke situation while plenty of ECs were still under hypoxia, which is impossibe for drug delivery, ROS accumulation and NETs formation. Consequently, the NETs forming ability can be underestimated especially in the model group. High intra‐group variability observed in several assays suggests that more refined methodologies or tighter experimental controls are needed to further substantiate these findings. Co‐treatment with Vc and DFOM markedly improved neuronal infarction, but failed to enhance the suppression of NETs formation in vivo. This suggests that their enhanced effects can be dependent on mechanisms beyond ROS and NETs that needs to be fully elucidated.

## 5 CONCLUSION

NETs formation on the endothelium under H/R is dependent on both ROS and extracellular iron. Combined administration of Vc and DFOM synergistically alleviated brain injury in MCAO mice, at least in part by reducing iron and ROS levels and inhibiting NETs formation. Given that DFOM and Vc are clinically available agents, and their repurposing in stroke treatment warrants further preclinical studies, especially with refined H/R models and longer‐term outcome evaluation.

## AUTHOR CONTRIBUTIONS

J.P. and R.Z. conceptualized and designed the present study and provided administrative support. W.F., Z.F., and Y.W. made contributions to the acquisition of data, and were involved in drafting the manuscript. T.Z. and K.H. analyzed and interpreted the data. J.P. and R.Z. critically revised the manuscript for significant intellectual content. The final manuscript was read and approved by all authors.

## CONFLICT OF INTEREST STATEMENT

The authors declare no conflicts of interest.

## ETHICS STATEMENT

All animal experiments complied with GB/T 35823‐2018 and were approved by the Ethics Committee of the First Affiliated Hospital, College of Medicine, Zhejiang University (approval number: 2017SDKS092, approval date: February 15, 2017).

## Supporting information

Supporting Information S1

Table S1

## Data Availability

The data that support the findings of this study are available from the corresponding author upon reasonable request.
