# Supplementary material for: Iron enhances reactive oxygen species generation and initiates neutrophil extracellular traps formation on the endothelium to exacerbate stroke
Source: J Cell Commun Signal. 2025 Oct 3;19(4):e70050. doi: 10.1002/ccs3.70050 (PMC12494541; doi:10.1002/ccs3.70050)
Supplement: Supplementary file 1 — Supporting Information S1 [file CCS3-19-e70050-s001.docx]

Table S1. Fragments Per Kilobase of exon model per Million mapped fragments (FPKM), differentially expressed genes (DEGs) and Gene Set Enrichment Analysis (GSEA) enrichments for Gene Ontology (GO) terms and Kyoto Encyclopedia of Genes and Genomes (KEGG) pathways.
